# Integrated Temperature and Position Sensors in a Shape-Memory Driven Soft Actuator for Closed-Loop Control

**DOI:** 10.3390/ma15020520

**Published:** 2022-01-10

**Authors:** Johannes Mersch, Najmeh Keshtkar, Henriette Grellmann, Carlos Alberto Gomez Cuaran, Mathis Bruns, Andreas Nocke, Chokri Cherif, Klaus Röbenack, Gerald Gerlach

**Affiliations:** 1 Institute of Solid-State Electronics, Faculty of Electrical and Computer Engineering, Technische Universität Dresden, 01062 Dresden, Germany; Gerald.Gerlach@tu-dresden.de; 2 Institute of Control Theory, Faculty of Electrical and Computer Engineering, Technische Universität Dresden, 01062 Dresden, Germany; Najmeh.Keshtkar@tu-dresden.de (N.K.); Klaus.Roebenack@tu-dresden.de (K.R.); 3 Institute of Textile Machinery and High Performance Material Technology, Faculty of Mechanical Engineering, Technische Universität Dresden, 01062 Dresden, Germany; Henriette.Grellmann@tu-dresden.de (H.G.); Carlos_Alberto.Gomez_Cuaran@mailbox.tu-dresden.de (C.A.G.C.); Mathis.Bruns@tu-dresden.de (M.B.); Andreas.Nocke@tu-dresden.de (A.N.); Chokri.Cherif@tu-dresden.de (C.C.)

**Keywords:** soft robotics, shape memory alloys, fiber rubber composite, integrated sensors, active structure control

## Abstract

Soft actuators are a promising option for the advancing fields of human-machine interaction and dexterous robots in complex environments. Shape memory alloy wire actuators can be integrated into fiber rubber composites for highly deformable structures. For autonomous, closed-loop control of such systems, additional integrated sensors are necessary. In this work, a soft actuator is presented that incorporates fiber-based actuators and sensors to monitor both deformation and temperature. The soft actuator showed considerable deformation around two solid body joints, which was then compared to the sensor signals, and their correlation was analyzed. Both, the actuator as well as the sensor materials were processed by braiding and tailored fiber placement before molding with silicone rubber. Finally, the novel fiber-rubber composite material was used to implement closed-loop control of the actuator with a maximum error of 0.5°.

## 1. Introduction

Soft robotics research and industry application have been and likely will be expanding over the next years [1]. These soft robots offer advantages compared to usual rigid robots in their versatility, adaptability and resilience. Therefore, they have great potential in robotic surgery, wearable robots, or the exploration of irregular environments. Structures used in the field consist of soft materials, mainly elastomers, in combination with actuator materials [2].

These elastomers can be reinforced by textiles, which may possess a higher stiffness in the fiber direction, but are still highly flexible. Such fiber-rubber composites are similar to systems found in nature like the combination of tendon fibers in soft tissue. In biomimetic structures, fibers may serve several functionalities, similar to tendons, nerve fibers, or muscle fibers in their biological role models. For example, Laschi et al. created an octopus inspired system [3]. Nerve fibers and muscle fibers correspond to the integrated sensors and actuators in soft robotic structures, respectively [4]. Accordingly, the reinforcement textile can be employed as stabilization to restrict unwanted motion patterns or may be equipped with electroactive [5] or thermoactive [6] actuator fibers to enhance their functionality.

Potential actuator mechanisms range from pneumatics [7], dielectric elastomers [8], or twisted carbon nanotube yarns [9] to shape memory alloys (SMAs) [10]. However, pneumatically powered soft actuators require bulky and heavy compressors, dielectric elastomer actuators have low force output and carbon nanotube yarns require an ionic liquid surrounding them. Therefore, in this work, SMAs are used due to their high force output, energy density, silent operation and good textile processability in wire form [11]. SMAs actuating mechanism is based on temperature induced phase transformation between martensite and austenite. An often-used SMA is a nickel-titanium compound (Nitinol) that contracts approximately 8% upon heating. The temperature increase necessary to activate the SMA is generated by passing an electrical current through the SMA, also known as Joule heating.

Due to inhomogeneous Joule heating or uneven convective cooling, a non-uniform temperature distribution might occur, resulting in localized stress. If the SMA wire were fixed along its length, that would lead to uneven and unwanted motion patterns and possible damages to the composite. Therefore, for higher performance, the SMA wire should be able to slide inside the soft matrix, which can be enabled by a core-sheath setup. As previously shown, with this approach large deformations can be achieved [12,13].

Another promising approach are bi-stable structures with high current input and thus high strain rate [14]. However, the full potential of soft robots can only be reached if they are able to operate continuously and autonomously in closed-loop control [15,16] and high strain rates require fast feedback and robust algorithms [17]. To enable the desired continuous motion and overcome the limits of bi-stable structures, feedback by sensors is required. This necessary feedback about the position and state of the structure is ideally recorded by integrated sensors [18]. Such sensors can be the actuator itself, e.g., in fluidic actuators [19], or additional piezoresistive yarns [20].

Using the SMA itself as a sensor itself is feasible but only within the phase transition or in its superelastic state [21]. The temperature-resistance relation of the SMA is non-monotonic outside of the change from martensite to austenite, which makes an additional temperature sensor necessary. Therefore, for SMA-actuated soft structures not only monitoring the position is essential, but also the temperature. In addition, the excessive temperature of the SMA wire can damage the surrounding structure and, additionally, drastically decrease the shape memory effect [22]. Consequently, the reliability and functional lifetime of such structures benefit from temperature monitoring. Previous works used external thermocouples [23] or thermistors attached to the SMA in non-soft systems [24]. However, this is diametrical to the soft-robotic approach. Currently, there is no soft material that incorporates SMA actuators in a core-sheath set-up and integrated, compliant sensors for temperature monitoring.

To overcome the discussed limitations of previous approaches, in this work Ni-Ti SMA wires are incorporated as a core in a braid. The braid has two main functionalities: (i) providing a sliding channel and (ii) measuring the temperature close to the SMA. This braid is then embroidered onto a glass-fiber reinforcement textile.

Moreover, the contraction to temperature relation in SMAs exhibits a strong hysteresis. Therefore, besides temperature monitoring, additional sensors measuring the position of the soft actuator are advantageous. Positional self-sensing has been demonstrated with various approaches ranging from commercial flex-sensors [25], which have the disadvantage of close-to-zero stretchability [26] to more advanced techniques like liquid metal strain gauges [27]. These liquid metal strain gauges are also a promising option for position self-sensing but simultaneously have several drawbacks like difficult processability, high cost, restricted temperature range and potential leakages [28]. In contrast, conductive yarns can be easily processed with textile technologies, and are cheap and robust [29]. They can be highly stretchable [30] and very strain sensitive [31]. However, such conductive fibers or yarns may have relatively high stiffness and exhibit hysteretic or non-monotonic strain-resistance behavior. These properties can be improved by converting the conductive fibers into a braid.

To provide self-sensing capability to the soft actuator, a stretchable sensor braid with conductive yarns is added and molded with silicone rubber. A 3D-printed mold gives the soft actuator two soft hinges, which can be activated and monitored by two SMA braids and two sensor braids. For validation, the position of the soft actuator is tracked optically and compared to the synchronized signals of the temperature and strain sensor. Thereby, the feasibility of the developed sensor-actuator fiber-rubber composite material is evaluated. Finally, the integrated sensors are used to control the position of the presented soft actuator by adjusting the current passing through the actuator with a PID controller.

This is the first presentation of a completely soft, fiber-based material, which features actuation and self-sensing of position and temperature for closed-loop control.

## 2. Materials and Methods

**Figure 1:**
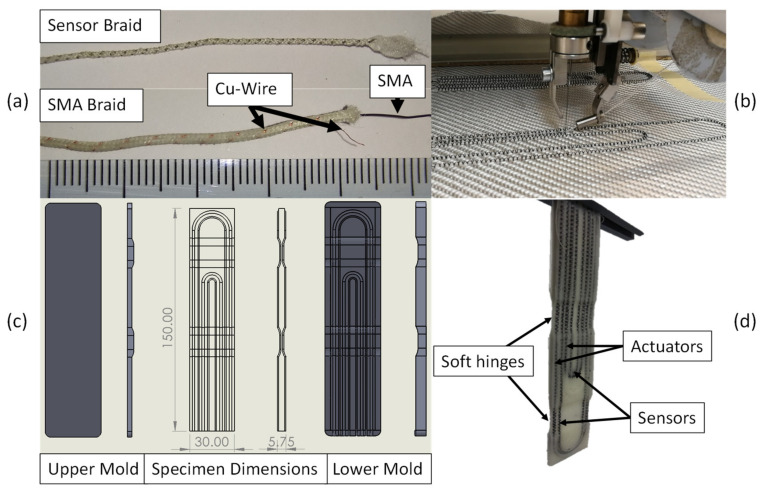
Manufacturing process of the soft actuator from (**a**) a braided Ni-Ti SMA core-sheath structure with copper wire and sensor braid; (**b**) tailored fiber placement of the braids to apply them to the glass fiber reinforcement textile; (**c**) molds and dimensions of the specimen; and (**d**) manufactured specimen with two soft hinges, two actuator and two sensor braids.

The production of the specimen was divided into the following steps that are illustrated in Figure 1:

1. Overbraiding the SMA wire with copper and polyamide yarns.
2. Tailored fiber placement of the braid on a glass-fiber woven.
3. Tailored fiber placement of silver-plated yarn braids on the same textile.
4. Molding the textile with silicone to form the soft actuator.
5. Extracting and removing insulation from wires and conductive yarns.

The SMA wire was a 0.3 mm diameter, high-temperature variant of the flexinol wire (Dynalloy Inc., Irvine, CA, USA). The SMA was a Nitinol (Nickel-Titanium) alloy with a transition temperature between 70 and 90 °C. An RU 2/12-80 braiding machine (August Herzog Maschinenfabrik GmbH & Co. KG, Osnabrück, Germany) was used for the production of the braided actuators and sensors. Eleven 312 dtex polyamide 6.6 yarns (W. Barnet GmbH & Co. KG, Aachen, Germany) and an AWG36 (0.12 mm diameter) insulated copper wire (J. G. Dahmen GmbH & Co. KG, Iserlohn, Germany) were braided around the Ni-Ti SMA wire. The insulation coating of the copper wire prevents an electrical connection between the copper and Ni-Ti wires. Figure 1a shows the produced actuator and sensor braids.

An important parameter is the braiding density. It should be as high as possible, so the braid forms a tube around the SMA wire. Then the SMA wire can slide inside the braid with only little frictional resistance. Thus, the braiding density was iteratively increased until 15 braids/cm, which was the highest, in a stable process, achievable density. Higher densities lead to the braiding yarns colliding with the braiding machine. In contrast, lower densities or a half-loaded braiding machine that would allow faster production speeds, lead to breakages of the SMA wire after activation due to the wires’ restricted motion in the matrix.

Afterward, the braid was evaluated with regard to its temperature–resistance behavior. An infrared camera (FLIR E95, FLIR Systems, Wilsonville, OR, USA) was used to track the temperature of a 100 mm sample of the braid, while the SMA core was activated by a power source (R&S HMP4040, Rohde & Schwarz GmbH & Co. KG, München, Germany) at different voltages. To calculate the temperature of the braided sheath from the thermographic images, the emissivity of polyamide (0.94) was used. The resistance of the copper wire was measured using a precision multimeter (Keithley DAQ6500-7700, Keithley Instruments, Cleveland, OH, USA) in a four-wire setup. This four-wire setup is necessary because of the low base resistance (<1 Ω) of the copper wire. By using the four-wire setup the resistances of the cables between multimeter and wire as well as the contact resistance between clips and wires can be eliminated.

The braid was then applied to a glass fiber 2 × 2 twill weave with a thickness of 0.18 mm (VR35, Lange + Ritter GmbH, Krefeld, Germany) using tailored fiber placement (type SGY 0200-650D by ZSK Stickmaschinen GmbH, Krefeld, Germany) with a 27 dtex polyester binder yarn. In order to improve stability during activation and prevent unwanted torsion, the reinforcement textile was rotated by 45°. A similar principle is used in the production of recurve bows [32]. As a result, the glass fibers in the weave decrease torsional instability in the solid body joint and restrict motion to bending. The sensor braid was produced according to [33], which includes the description of the strain-resistance relation of the variant HC 40 with 25 braids/cm that was used in this work.

A 3D-printed mold was used to produce a specimen of 150 mm length and 30 mm width. Grooves in the mold (see Figure 1c) ensured the precise location of the actuator and sensor braids. For the actuator and sensor braids a groove diameter of 1.5 mm, and respectively 0.9 mm, were used. The mold was then sealed on three sides with silicone glue and filled with elastomer (TFC Silicone Type 11 Shore A 22, TFC Silikonfabrik, Riede, Germany). After curing for 24 h at room temperature, the sample was removed from the mold and excess silicone cut. To contact the SMA and copper wire, the braid was opened at the ends as shown in Figure 1a. The SMA wires are fixed and contacted in luster terminals and the copper wires/conductive yarns with crimp connectors.

The electrical characterization and control experiments were carried out using the same R&S power source and Keithley multimeter as before. Both sensor signal and actuator output are linked by a Simulink program, which includes a PID control scheme. Before the actual experiments were performed, the soft actuator was run through several thermal loading cycles to stabilize its behavior.

**Figure 2:**
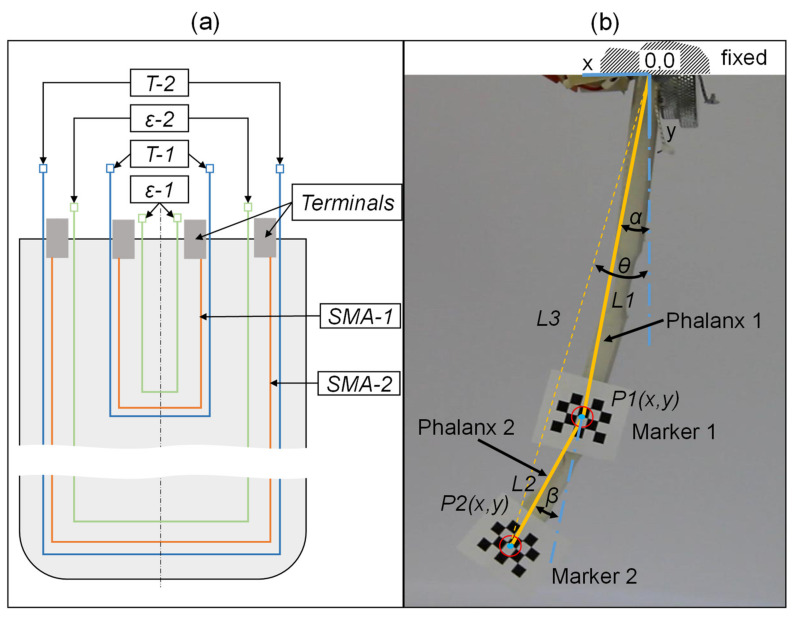
(**a**) Overview of the integrated sensors and actuators with copper wires as temperature sensors (T), braided silver-plated yarn as strain sensors (ε) and shape memory alloy wires (SMA) covering the first phalanx (1) and both first and second phalanx (2); (**b**) metrics to describe the phalanges position and configuration.

Figure 2a gives an overview of the sensors and actuators in the sample and their connections. The copper wire temperature and silver-plated braid strain sensors will be abbreviated as T-Sensor and ε-Sensor, respectively. The actuators and sensors in the smaller loop, which only spans over the first phalanx, are denoted as T-1, etc. while the ones in the larger loop over the whole sample will be denoted as T-2, etc.

In addition to the copper wires’ resistances, the multimeter recorded the sensor braids’ resistances as well as current and voltage applied to the SMA wires. From the latter two, the resistance of the SMA wire and the power input can be derived. Simultaneously, the motion of the soft actuator was tracked by using checkerboard markers and a digital single-lens reflex camera (Canon EOS 7D, Canon K-g, Tokyo, Japan). The lens distortion and pixel to length calibration as well as the tracking of the optical markers in the video was implemented in Matlab. Figure 2b shows the sample with attached optical markers and explains how the metrics of displacements were derived. After the pixel-to-length calibration, the positions of the two markers were available in x-y-coordinates with the origin being the end of the fixing clamp. From the x-y-coordinates of the markers, the angles α and β can be calculated. The angle between the y-axis and phalanx 1 is called α and the angle between phalanges 1 and 2 is called β. These metrics were used from here on because they allow an independent description of the first and second phalanges’ positions. When the first SMA wire is activated, the system should move around the first joint, so α changes but β does not. However, the x-y-position of the second marker changes although the second SMA is not activated. Therefore, the angles α and β are a better suited metric than Cartesian coordinates.

## 3. Results and Discussion

First, the results of the activation tests of the unembedded SMA braid are presented. After that, the results of the activation tests of the soft actuator are described and, finally, the results of the control experiments are shown.

### 3.1. SMA Braid

**Figure 3:**
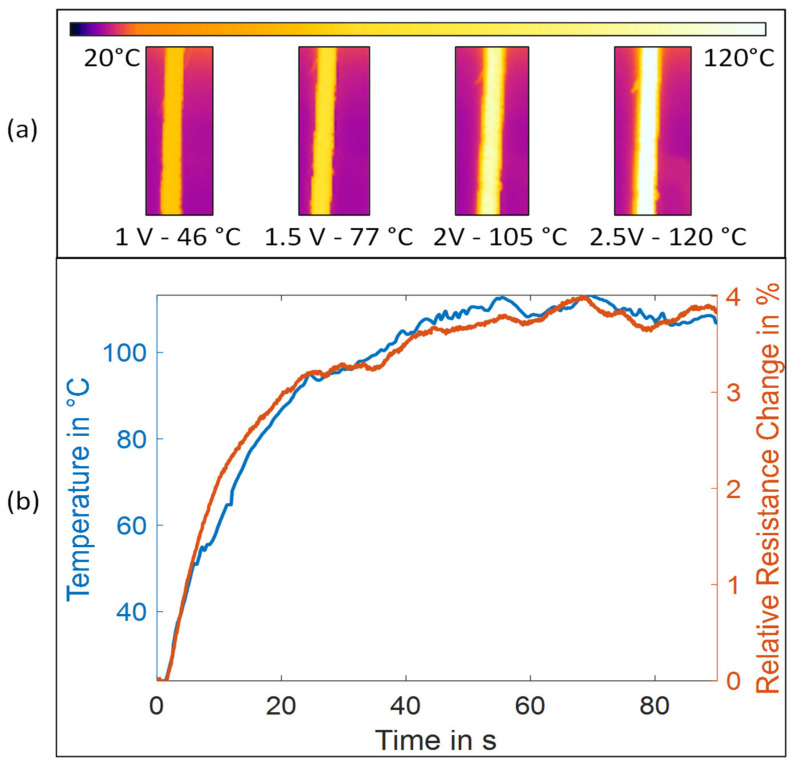
Evaluation of the braided sheath as a temperature sensor: (**a**) SMA braid at varying activation voltage after reaching steady state; and (**b**) relation between temperature and relative resistance change of the copper wire for the activation run with 2 V.

Figure 3 shows the temperature of the SMA braid during activation with 2 V and ~1800 mA as well as the relative change in resistance of the copper wire in the braided sheath. Temperature and resistance change are in good agreement with only slight deviations.

Moreover, considering the resistance temperature coefficient of copper (0.0039 K^−1^), the theoretically calculated resistance change and the measured resistance were also in good agreement. The maximum difference was 1.1 K.

Hence, the copper wire in the sheath is a feasible way to track the temperature of the actuator. However, there might be a difference between the temperature on the surface of the SMA braid and the SMA itself in the core. This factor has to be considered in the evaluation of the system and additionally depends on the environment of the soft actuator, i.e., forced or unforced convection.

### 3.2. Soft Actuator System

**Figure 4:**
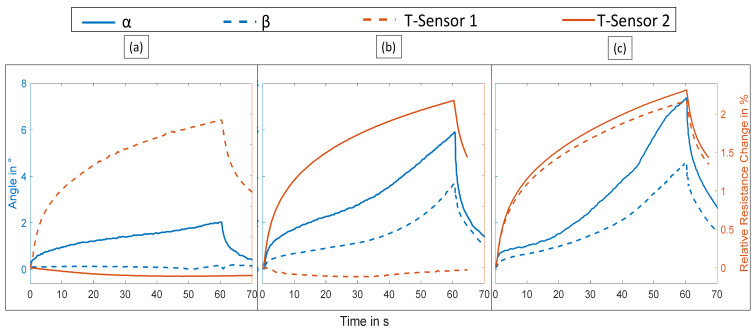
Deformation and resistance change of the temperature sensor in the braided sheath when a current of 1.1 A was run through the SMA wires for 60 s: (**a**) SMA wire 1 activated; (**b**) SMA wire 2 activated; (**c**) SMA wires 1 and 2 activated.

The presented soft actuator system was activated at varying electric currents between 0.6 and 1.4 A for 60 s. An exemplary video of the activation experiments is included in Supplementary Material Video S1. Figure 4 shows the results for an activation current of 1.1 A. First, the deformation of the actuator, which is represented by the two angles, is compared to the resistance change of the temperature sensors. For that, only SMA 1 is activated, then only SMA 2 is activated and finally, both are activated simultaneously. If only the first SMA is heated actively, the soft actuator only moves around its first hinge, i.e., the angle α, as expected. Both β and the signal of T2 remain near zero because the second phalanx does not move relative to the first phalanx and the temperature of SMA 2 does not increase.

In contrast, if only SMA 2 is activated, α and β both increase but the signal of T1 is stable. This is also to be expected as SMA 2 spans both phalanges. Figure 4c shows that upon activation of both SMA wires, the deformation increases in comparison to the single wire activation runs and the resistance of both temperature sensors changes accordingly.

Consequently, the use of the two temperature sensors allows individual monitoring of both SMA actuators. Even though the resistance increase is higher if both actuators are heated, this assessment does not change. In that case, both SMA wires and their sheaths, indeed reach comparably higher temperatures because of the heat transfer between the two wires. Nevertheless, there is a small difference between temperature sensors 1 and 2 for the same activation current, which is likely due to a slight variation in heat transfer to the environment.

Moreover, the time delay between activation and sensor signal was analyzed. The time difference between the current in the SMA and the resistance of the temperature sensor amounted to 0.15 s, which corresponds to the switching frequency of the multimeter. This means that the rise time is below 0.15 s, sufficiently fast for closed-loop control considering the relatively low actuation frequency (<1 Hz) used for SMA-based actuators.

**Figure 5:**
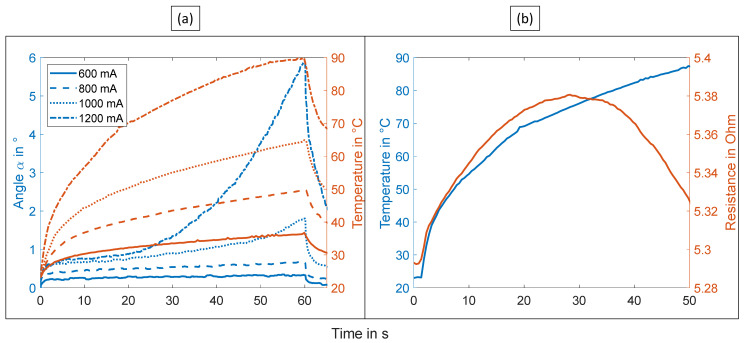
(**a**) Deformation and temperature measured by the integrated temperature sensor at different currents; and (**b**) resistance of the SMA wire during heating versus temperature measured with the integrated temperature sensor.

As Figure 5a illustrates, the temperature and, based on the actuator mechanism of SMAs, the deformations increase with rising activation currents.

Therein the temperature was calculated from the temperature coefficient of resistance *κ*, the relative resistance change Δ*R*/*R* and the room temperature *T_room_* by:

*T* = *κ*^−1^ ∗ Δ*R*/*R* + *T_room_*. (1)

As expected, higher currents lead to higher temperatures and higher deformations. According to the manufacturer’s datasheet, the phase transition temperature lies between 70 and 90 °C (+/− 10 °C). Because the phase transition only occurs above ~70 °C, the actuator only showed larger deformations with activation currents over 1 A. Below that, only a small amount of motion was observed.

However, it has to be emphasized that this makes the internal resistance of the SMA wire difficult to use for closed-loop control. Figure 5b shows the relation between temperature and resistance during activation. The resistance signal of the heated SMA wire is non-monotonic. Before reaching the phase transition, the temperature coefficient of resistivity is positive as it is for most metals. During the phase transition, the resistance decreases because the phase transition effect dominates the resistance change. The local maximum around 70 °C coincides with the expected start of the phase transition. This behavior was already described in more detail in previous publications [34]. Theoretically, the internal resistance of the SMA can be used to control deformation. However, even with novel techniques like machine learning, the results are not convincing because of the challenge posed by the inherent non-monotonicity [35]. Therefore, the resistance of the SMA wires was not used for the control experiments in this work.

**Figure 6:**
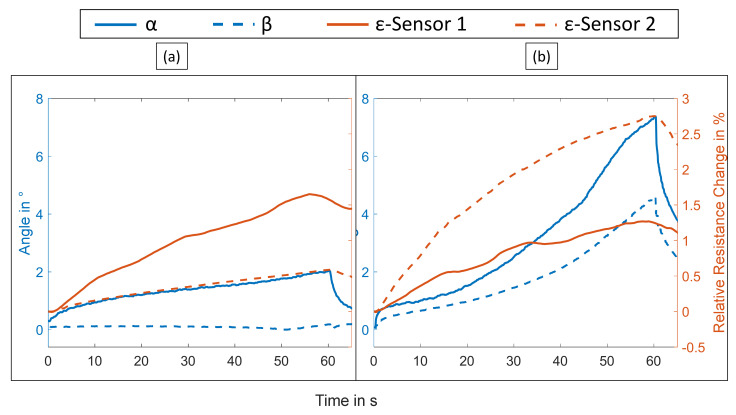
Deformation and resistance change of the strain sensors when a current of 1.1 A was run through the SMA wires for 60 s: (**a**) SMA wire 1 activated and (**b**) SMA wires 1 and 2 activated.

Figure 6 shows the resistance signals of the braided strain sensors when the SMA wires are activated. In contrast to the temperature sensor, which reacted nearly exclusively to the SMA wire they surround, the strain sensors both reacted even if only SMA 1 is activated. That is sensible because both strain sensors cover the first joint. If both SMA wires were activated and, therefore, both soft hinges were rotated, the second strain sensor’s signal was higher because it was strained significantly more. Overall, the signals of the integrated strain sensor were in good correlation with the deformation of the soft actuator. As can be seen also in Figure 6, the signal of strain sensor 1 was less stable than that of sensor 2. This might be due to slippage of the sensor in the matrix or other fabrication-related irregularities, such as exalted fiber tension during the tailored fiber placement process. In addition, the signal did not recover its initial value as fast as the deformation would indicate. This was also not the case for the temperature sensors. The cause may be that the strain sensor is cross-influenced by the overall temperature increase of the soft actuator. Because the strain sensors are positioned further away from the SMA wires than the temperature sensors, their temperature increases and decreases less but are also delayed. As a result, the signal is a superposition of temperature and strain, which makes some kind of compensation of the temperature influence necessary.

### 3.3. Closed-Loop Control

Finally, the integrated temperature sensors were used for closed-loop control because they offer the most stable and reliable signal of the three available sensor types. First, the sensor signal of the second temperature sensor that corresponded to deformations of 1°, 8° and 6° was calculated from the previous experiments. These were fed into a Simulink PID block, which compared the setpoint to the measured temperature signal and adjusted the current output accordingly (PID parameters, manually tuned: *P* = 250, *I* = 10, *D* = 2). The PID parameters were chosen with favoring stability over velocity. Therefore, the response is relatively slow both while cooling and heating.

**Figure 7:**
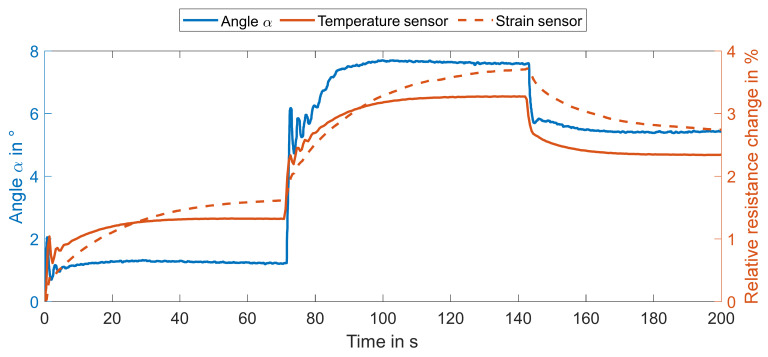
Deformation and signals of temperature and strain sensors 2 during the control experiments for activation of SMA wire 2 based on a PID controller.

Figure 7 illustrates the results of the control experiment. Most importantly, the deformation, as well as both sensor signals, followed the desired step function with three plateaus quite well. In addition, in the first section, both the temperature signal, as well as the angle was very close to the setpoints after a short settling phase.

In the second and third sections, the difference between the temperature signal setpoint and actual value was close to zero. However, there was a deviation of 0.5° from the desired angles of 8 and 6°. The deviation is caused by the hysteretic nature of the SMA and is amplified by the non-linear relation between temperature and strain. Nevertheless, the signal of the integrated temperature sensor is feasible to control the soft actuator. It is better suited than the internal resistance of the SMA wires and, additionally, can prevent overheating and resulting damages to SMA wires and overall structure. Because the compliant nature of the soft actuator allows for lower control precision in comparison with traditional industrial robots, the signals of the integrated sensors are sufficient for target applications like handling, i.e., gripping or pushing, delicate materials, such as soft tissue or berries. For this purpose, multiple soft actuators can be combined to form a complex, highly versatile system, which may be paired with other faster actuator mechanisms, e.g., dielectric elastomers, for better performance.

However, several other aspects deserve further research. Especially the long-term stability of the whole composite but also the individual components requires evaluation. In the presented work, the soft actuator was activated at different current levels for a total time of more than 50 h with no notable change in performance or sensitivity. Still, for real-world applications more rigorous testing with several thousand cycles needs to be carried out to assess sensor drift, aging, or delamination. In addition, also the application under different environmental conditions should be analyzed. That applies to both thermally variable or corrosive environments. Specifically, a non-uniform temperature distribution or non-uniform convection might impact performance drastically even though the negative temperature coefficient in the transition phase results in a self-regulating system.

Moreover, the use of non-silicone rubbers can improve performance and stability under certain conditions, especially with regard to aging and the thermal stress caused by the SMA activation.

## 4. Conclusions

In summary, a soft actuator was presented that is based on a novel fiber-rubber composite material. This fiber-rubber composite includes a reinforcement textile with shape memory wire actuators, as well as strain and temperature sensors. The temperature sensors are incorporated in a newly developed braid surrounding the SMA wire and allow for precise temperature monitoring.

Based on the temperature sensor signal, the temperature and thereby the strain of the SMA wires can be controlled in closed-loop mode. Stability, speed and precision may be improved by using more advanced control algorithms, such as robust control [36]. Moreover, taking the hysteresis of the temperature-strain relation into account would further improve the performance.

Furthermore, the consideration of both strain and temperature sensors in a complex control algorithm might further improve precision and additionally compensate for outside perturbations. Therefore, the braided strain sensor should be developed further with regard to their temperature cross-sensitivity by either material development or compensation techniques.

Finally, the developed fiber-rubber composite material can be used in more complex soft robots with more degrees of freedom and evaluated in diverse application scenarios like locomotion in uneven terrain or gripping and handling sensitive materials.

## Data Availability

Data is available from the authors open reasonable request.

## Supplementary Materials

The following supporting information can be downloaded at: https://www.mdpi.com/article/10.3390/ma15020520/s1, Video S1: Activation experiment of the soft actuator.
